# Evaluation of the Antioxidant and Anti-Lipoxygenase Activity of *Berberis vulgaris* L. Leaves, Fruits, and Stem and Their LC MS/MS Polyphenolic Profile

**DOI:** 10.3390/antiox12071467

**Published:** 2023-07-21

**Authors:** Anna Och, Marta Olech, Kamil Bąk, Sebastian Kanak, Anna Cwener, Marek Cieśla, Renata Nowak

**Affiliations:** 1 Department of Pharmaceutical Botany, Medical University of Lublin, 1 Chodźki St., 20-093 Lublin, Poland; 2 Botanical Garden, Maria Curie-Skłodowska University in Lublin, 3 Sławinkowska St., 20-810 Lublin, Poland; 3 Institute of Medical Sciences, Medical College of Rzeszow University, 35-025 Rzeszow, Poland

**Keywords:** *Berberis vulgaris*, antioxidants, phenolic acids, flavonoid glycosides, flavonoid aglycones, anti-lipoxygenase

## Abstract

*Berberis vulgaris* L. is currently widely studied for its antioxidant and chemopreventive properties, especially with regard to the beneficial properties of its fruits. Although the bark and roots have been well known and used in traditional medicine since ancient times, little is known about the other parts of this plant. The aim of the research was to determine the antioxidant and LOX inhibitory activity effects of extracts obtained from the leaves, fruits, and stems. Another aim of the work was to carry out the quantitative and qualitative analysis of phenolic acids, flavonoid aglycones, and flavonoid glycosides. The extracts were obtained with the use of ASE (accelerated solvent extraction). The total content of polyphenols was determined and was found to vary depending on the organ, with the highest amount of polyphenols found in the leaf extracts. The free radical scavenging activity of the extracts was determined spectrophotometrically in relation to the DPPH (2,2-diphenyl-1-picrylhydrazyl) radical, with results ranging from 63.9 mgTE/g for the leaves to 65.2 mgTE/g for the stem. Antioxidant activity was also assessed using the ABTS test. The lowest value was recorded for the barberry fruit (117.9 mg TE/g), and the highest level was found for the barberry leaves (140.5 mgTE/g). The oxygen radical absorbance capacity test (ORAC) showed the lowest value for the stem (167.7 mgTE/g) and the highest level for the leaves (267.8 mgTE/g). The range of the percentage inhibition of LOX was determined as well. The percentage inhibition of the enzyme was positively correlated with the sum of the flavonoids, TPC, TFC, and the content of selected flavonoids. Phenolic acids, flavonoid aglycones, and flavonoid glycosides were determined qualitatively and quantitatively in individual parts of *Berberis vulgaris* L. The content of phenolic acids, flavonoid aglycones, and flavonoid glycosides was determined with the LC-MS/MS method. The following phenolic acids were quantitatively and qualitatively identified in individual parts of *Berberis vulgaris* L.: gallic acid, 3-caffeoylquinic acid, protocatechuic acid, 5-caffeoylquinic acid, 4-caffeoylquinic acid, and caffeic acid. The flavonoid glycosides determined were: eleutheroside E, Eriodictyol-7-glucopyranoside, rutin, hyperoside, isoquercitin, luteoloside, narcissoside, naringenin-7-glucoside, isorhamnetin-3-glucoside, afzeline, and quercitrin. Flavonoid aglycones such as catechin, luteolin, quercetin, and eriodictyol were also determined qualitatively and quantitatively.

## 1. Introduction

*Berberis vulgaris* L. (*Berberidaceae*) is a medicinal plant of the genus *Berberis*. It is currently becoming increasingly popular among scientists looking for new sources of antioxidants, chemopreventive agents, and nutraceuticals. Currently, the species is highly valued in the Middle East, mainly in Iran, which is the main producer of barberry fruit [1] for culinary purposes. In recent centuries, *Berberis vulgaris* L. has also been widely used in traditional medicine in Europe. It was used mainly for its content of berberine and its beneficial effects, e.g., in diseases of the liver and cardiovascular system. Unfortunately, for agricultural reasons, this species has been eradicated in Europe as it is an intermediate host of stem rust—a parasite of cereals. Currently, research on *Berberis vulgaris* L. has generated renewed interest in this species, mainly in its valuable fruit, which is a source of nutrients and a chemopreventive agent. The studies on total polyphenol content and antioxidant activity have so far focused on barberry fruit extracts, excluding the other parts of the plant [2,3]. However, the increasing popularity of *Berberis vulgaris* L. is also related to its anticancer effect [4,5,6,7,8,9,10].

Despite the promising results obtained for selected barberry species, relatively few of them have been tested with respect to their chemical composition, chemopreventive potential, and nutraceutical use. The content of polyphenols and flavonoids and the antioxidant properties have been determined only for selected *Berberis* L. species. For example, the high content of polyphenols in the herb, roots, and fruits of *Berberis cretica* [11] and the aerial parts of *Berberis sibirica* [12], with the highest antiradical activity depending on the solvent used, was 40 µg/mL. The TPC value of 190 mgGAE/g was determined for *Berberis cretica* (with an antiradical activity of IC50 = 60 µg/mL), and the TPC value of 159 mgGAE/g was reported for *Berberis sibirica* [11,12]. In fresh *Berberis heteropoda* fruit, the TPC value was at the level of 68.55 mgGAE/g, expressed as milligrams of gallic acid equivalent per gram of fresh fruit mass, and the TFC value was 108.42 mgQE/g, denoted as milligrams of rutin equivalent per gram of fresh fruit mass [13]. In turn, the evaluation of the potential benefits of the *Berberis nummularia* and *Berberis atrocarpa* fruits showed that the TPC and TFC were relatively lower than those in the other barberry species, with the values of *Berberis nummularia* at the levels of 2 mg GAE/g, expressed as mg of gallic acid equivalents/g of dry extract, and 2 mg RE/g, expressed as mg of rutin equivalents/g of dry extract, respectively. For *Berberis atrocarpa*, TPC was established at the level of 12 mg GAE/g, expressed as mg of gallic acid equivalents/g of dry extract, and TFC at the level of 9 mgRE/g, expressed as mg of rutin equivalents/g of dry extract [14]. The chemopreventive and nutraceutical potential of *Berberis orthobotrys* Bienert ex c.k. Schneider has also been demonstrated. The highest values were demonstrated for the extracts obtained with the use of 80% methanol: TPC 53.86 mgGAE/g, expressed as mg of gallic acid equivalents (GAE) per g of dried extract, and TFC 13.94 mgCE/g, expressed as mg of quercetin equivalents (QE) per g of dry extract [15]. The valuable properties of *Berberis thunbergii* DCs have also been demonstrated, with the highest values for extracts obtained with 80% methanol, i.e., TPC 216 mg GAE/g, expressed as mg of gallic acid equivalents/g of dry extract, and TFC 46 mg RE/g, expressed as mg of rutin equivalents/g of dry extract. The antioxidant activity of *Berberis thunbergii* DC leaves was determined to be at the levels of DPPH 429 mg TE/g, expressed as mg of Trolox equivalents/g of dry extract, and ABTS 450 mgTE/g, expressed as mg of Trolox equivalents/g of dry extract [16]. 

The antioxidant activity of *Berberis vulgaris* L. has so far been examined only partially and, as already mentioned, the literature data are focused on the fruits and show that the results largely depend on the extraction method, plant growing habitat, and varieties. The current data usually come from Iran and Turkey, and the review of these literature data has shown that the extraction with 80% methanol yields extracts with the highest antioxidant potential. So far, only some studies have been focused on the antioxidant potential of other plant organs. For example, Gorizpa et al., 2022 tested the usefulness of the dried ethanol root and bark extracts of *Berberis vulgaris* L. subsp. *asperma* and *orientalis* as natural preservatives, showing high antioxidant activity [17]. El-Zahar et al., 2022 assessed the antioxidant activity of leaves and root extracts and determined the phenolic and flavonoid composition quantitatively and qualitatively [18].

**Figure 1:**
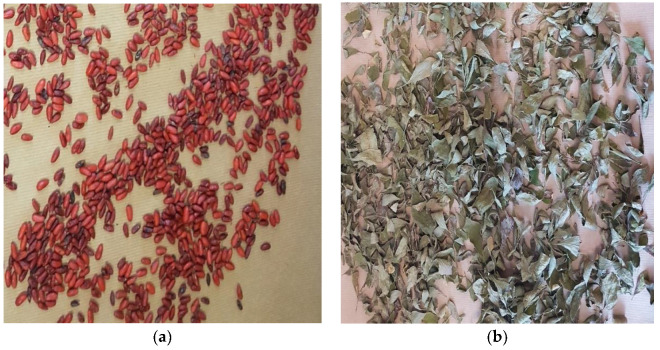

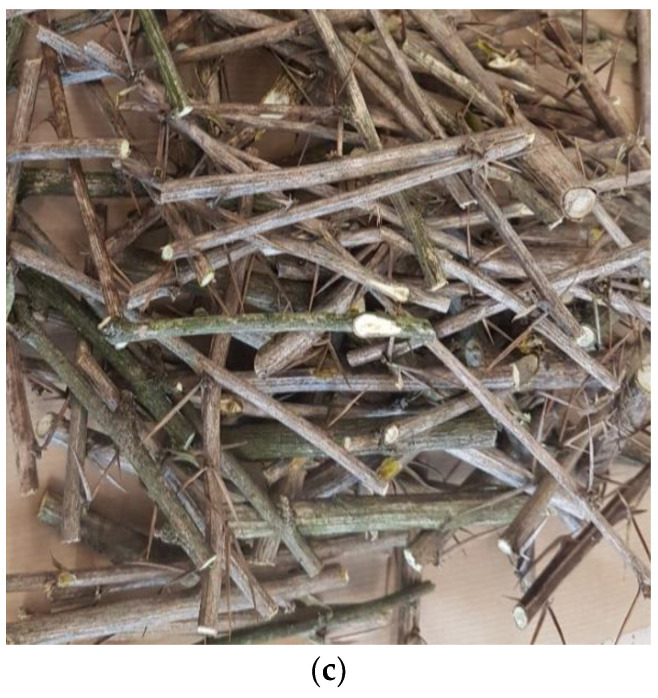
Dried parts of plant material obtained for investigations: (**a**) fruits, (**b**), leaves, and (**c**) stem.

However, the data on the quantity and quality of *Berberis vulgaris* L. secondary metabolites are incomplete and mostly refer to the fruit. It is known to be a very rich raw material, but which secondary metabolites are responsible for the antioxidant properties is still only partially known. The plant is phytochemically rich, and various groups of compounds may be responsible for the activity. Tannins, anthocyanins, stilbene derivatives, and triterpenes are very important from this point of view. Because flavonoid glycosides, flavonoid aglycones, and phenolic acids represent very important groups of relationships from this point of view, this work attempts to determine their quantitative and qualitative contents. The purpose of the study was to analyse the qualitative composition of polyphenols and flavonoids as well as the antioxidant and LOX inhibitory effects of extracts obtained from the fruits, leaves, and stem of *Berberis vulgaris* L. (Figure 1). We also analysed the qualitative and quantitative content of individual phenolic acids, active flavonoid aglycones, and flavonoid glycosides. In this article, the antioxidant activity of the stem, leaves, and fruits was assessed using three different methods of antioxidant capacity: the DPPH, ABTS, and ORAC methods. Similarly, the quantitative and qualitative analysis of the content of phenolic acids, flavonoid aglycones, and flavonoid glycosides, performed using the LC-MS method, was assessed in the stem, leaves, and fruits of *Berberis vulgaris* L. Among the flavonoid glycosides, we qualitatively and quantitatively determined eleutheroside E, rutin, luteoloside, isoquercetin, narcissoside, isorhamnetin-3-glucoside, quercitrin, naringenin 7-glucoside, and afzelin. We also evaluated the inhibitory activity of *Berberis vulgaris* L. against the inflammatory enzyme LOX and obtained interesting data showing that barberry appears to have anti-inflammatory potential. Although the anti-inflammatory activity of specific secondary metabolites of barberry has already been demonstrated, the effect on the LOX enzyme has not been evaluated so far. The research was carried out on all the organs of the plant growing in the Maria Curie-Sklodowska University Botanical Garden in Lublin; the organs were collected in September. This collection also provided information on the differences in the composition of the secondary metabolites in relation to the specimens previously studied in Turkey or Iran.

## 2. Materials and Methods

### 2.1. Materials and Reagents

The plant material of the *Berberis vulgaris* L. barberry stem, leaves, and fruits was obtained from the Maria Curie-Skłodowska University Botanical Garden in Lublin in September 2020 (Voucher specimen: AO2020091). The raw material was separated and dried at room temperature in the shade with ventilation. The raw material was weighed and ground in an electric mill and portioned, vacuum-packed, and stored in a closed package at −30 °C until the start of the tests. DPPH• (2,2-diphenyl-1-picrylhydrazyl), Trolox, gallic acid, 3-caffeoylquinic acid, protocatechuic acid, 5-caffeoylquinic acid, 4-caffeoylquinic acid, caffeic acid, catechin, luteolin, eriodictyol-7-glucopyranoside, quercetin, syringaresinol-di-O-glucoside, rutin, hyperoside, luteoloside, isoquercetin, narcissoside, isorhamnetin-3-glucoside, quercitrin naringenin 7-glucoside, afzelin, LC grade acetonitrile, Trolox, 2,2′-azino-bis (3-ethylbenzothiazoline-6-sulfonic acid) (ABTS•+), Folin–Ciocalteu reagent, and 2,2′-azobis (2-methylpropionamide) dihydrochloride (AAPH) were purchased from Sigma–Aldrich (Stenheim, Germany); ascorbic acid was purchased from Stanlab (Poland); methanol and aluminium chloride hexahydrate of analytical grade were purchased from POCH (Gliwice, Poland).

### 2.2. Sample Extraction and Process

A 2 g amount of powdered barberry stem, leaves, and fruits from *Berberis vulgaris* L. was extracted by accelerated solvent extraction (ASE). Accelerated solvent extractions with an 80% methanol concentration (3 cycles for 10 min each at 80 °C) were performed on an ASE 150 system from the Dionex Corporation (Sunnyvale, CA, USA). All extracts were prepared in triplicate. In all cases, the extracts obtained were evaporated to dryness under reduced pressure and lyophilised in a Free Zone 1 apparatus (Labconco, Kansas City, KS, USA). The residue was weighed and redissolved in the same solvent used for the extraction in order to obtain stock solutions with the appropriate concentration, which were stored in the refrigerator at −30 °C until the start of the tests. Samples of the plant extracts for testing were prepared immediately prior to analysis by dissolving them in an ultrasonic bath. A weighed amount of the extracts after lyophilization was dissolved in a measuring volume of 80% methanol to obtain starting solutions with a concentration of 40 mg/mL. As required for the determinations, they were diluted with the same solvent to a specific concentration.

### 2.3. Determination of Total Phenolic, Total Tannin, and Total Flavonoid Contents

The analysis of the total phenolic content (TPC) was carried out using the modified Folin–Ciocalteu method [19]. The TPC was determined using a standard curve prepared for gallic acid. The absorbance was read at 680 nm after a 20 min incubation. The results were expressed in mg of gallic acid per 1 g of dry weight of dry extract (mg GAE/g—gallic acid equivalent). The total flavonoid content (TFC) was determined according to the method proposed by Lamaison and Carret (1990) with modifications. The absorbance was measured at 430 nm after a 30 min incubation against a blank containing methanol instead of the test sample. The results were expressed in mg of quercetin (Q) per 1 g of dry extract.

The total tannin (TTC) content was determined using the vanillin/HCl method [20]. The absorbance was measured at 500 nm after a 20 min incubation against a blank containing methanol instead of the tested sample. The results were expressed in mg of pyrogallol (P) per 1 g of dry extract. The measurements were determined using 96-well transparent µplates (Nunclon. Nunc. Roskilde, Denmark) and an Infinite Pro 200F µplate reader (Tecan Group Ltd., Männedorf, Switzerland). 

### 2.4. Antiradical Activity Analysis

#### 2.4.1. Determination of Antiradical Potential with the DPPH• Assay

The antioxidant assay was carried out using 2,2-diphenyl-1-picrylhydrazyl (DPPH) and the method developed by Brand-Williams et al., with modifications [21,22]. Absorbance was measured after 60 min at 517 nm using an Infinite Pro 200F µplate reader (Tecan Group). The results were obtained from measurements made for each sample and expressed as Trolox equivalents—expressed as mg Trolox of dry extract [mgTE/g].

#### 2.4.2. Determination of Antiradical Capacity with the ABTS•+ Assay

The antiradical activity was determined using the refined ABTS+• discolouration test, with modifications [23,24]. The absorbance was measured at 734 nm after a 6-min incubation. The ability of the extract to quench ABTS+• free radicals was determined using Equation (1), as follows:

Capture % = [(AC − AA)/AC] × 100 (1)

where AC is the absorbance of the control and AA is the absorbance of the sample. The results were obtained from the measurements made for each sample and expressed as Trolox equivalents—expressed as mg Trolox of dry extract [mgTE/g].

#### 2.4.3. Oxygen Radical Absorbance Capacity (ORAC) Assay

The determination of the oxygen radical absorbance capacity (ORAC) was carried out according to a method developed by Huang et al. (2002) [22,25], with modifications. 

All assays were performed with an Infinite Pro 200F µplate reader in triplicate. The activity of the sample was expressed as Trolox equivalents—expressed as mg Trolox of dry extract [mgTE/g].

### 2.5. Lipoxygenase Inhibitor Screening Assay

The anti-lipoxygenase activity of the *Berberis vulgaris* L. extracts was determined by spectrophotometric evaluation of the inhibition of the LOX enzyme activity using the method described by Baraniak and Szymanowska [26]. It was calculated from the absorbance measured immediately at the wavelength of 234 nm. The samples were measured in triplicate. The percentage inhibition was calculated as follows:

% inhibition of LOX = (AK − AP)/AP × 100%

where AK means increased absorbance of the control and AP means increased absorbance of the sample.

### 2.6. Determination of Phenolic Acids, Flavonoid Glycosides, and Flavonoid Aglycones Using the LC-MS/MS method

The content of polyphenolic compounds was determined by liquid chromatography–mass spectrometry (LC-MS) according to the method developed by Łyko et al. (2022), with modifications [27]. The experiments were carried out using an Agilent 1200 Series LC apparatus (Agilent Technologies, Santa Clara, CA, USA) connected to a triple quadrupole mass analyser (3200 QTRAP; Sciex, Redwood City, CA, USA). The electrospray ionization (ESI) interface worked in the following conditions: temperature, 500 °C; curtain gas at 23 psi; source voltage in the nebulizer gas at 50 psi; and negative ionisation mode, 4500 V. The mass analyser was set to perform the analyses in the multiple reaction monitoring (MRM) mode. The separations were carried out on an Eclipse XDB-C18 column (4.6 × 150 mm, 5 μm bead diameter; Agilent Technologies, CA, USA) at 20 °C using the same chromatographic conditions as those described by Łyko et al., 2022 [27]. Data were acquired and processed with Analyst 1.5 software (Sciex, Redwood City, CA, USA). Optimised LC-MS settings were determined experimentally for each compound and are given in Table S1 in the Supplementary Material. Quantitative analysis was also performed in the multiple reaction monitoring mode based on the peak area of the most intense MRM transition of every identified analyte, and the results from the calibration curve were used to prepare its analytical standard. Standard curves were generated by three repeated injections of known concentrations of standard solutions. The optimised analytical parameters used for the quantitative determinations are given in Table S2 (Supplementary Material). The LOD (limit of detection) and LOQ (limit of quantification) values were established at signal-to-noise ratios of 5:1 and 10:1, respectively. All the experiments were performed in triplicate. The results were expressed as ng/mg of dry extract.

## 3. Results and Discussion

### 3.1. Polyphenol Content in Individual Organs of Berberis vulgaris

In our study, the determined amounts of polyphenols were found to have very similar levels: 58.5 ± 0.01 mg/g for the leaf extract; 57.7 ± 0.001 mg/g for the stem extract; and 52.8 ± 0.01 mg/g for the fruit extract. There are few data in the literature about the levels of polyphenols in organs other than the fruits of *Berberis vulgaris* L. El-Zahar et al., 2022 determined the TPC in the leaves of *Berberis vulgaris* L. at the level of 120.7 ± 1.2 mgGAE/g, expressed per g of dry extract [18]. According to the literature, the content of polyphenols in the fruits vary depending on the extract, from the level of 92.7 mg GAE per g of dry extract in the acetone extract; 49.9 mg GAE per g of dry extract in ethanolic extract; 48.9 mg GAE per g of dry extract in decoction; and 39.4 mg GAE per g of dry extract in infusion [2]. Mottaleb et al. showed TPC level in the fruit of *Berberis vulgaris* L. when using an 80% methanol extract, where the content of polyphenols was 280 mg GAE per g of dry extract. Simultaneously, they compared the level in a water extract which was at the level of 100 mg GAE per g of dry extract [28]. Dimitrijević et al., 2020 reported that the TPC in wild fruits was at the level of 494 ± 2 mg GAE per g of dry methanolic extract [29]. Eroğlu et al., 2020 described the TPC of barberry dry fruits, with the lowest level in a water extract [148.0 ± 30.3 mgGAE/g dry fruits] and the highest content in an ethanol extract [448.3 ± 81.2 mgGAE/g dry fruits] [3]. Khromykh et al., 2018 determined the TPC at the level of 10.5 ± 54.3 mgGAE/g of the fresh weight [30]. Okatan et al., 2018 described total phenol contents ranging from 11.9 to 26.2 mg GAE/g of the fresh weight, depending on the plant variety [31]. The values of the TPC in the fruits analysed in our study were very similar in each part of the plant investigated by us and ranged between 52.8 ± 0.01 mg of gallic acid per 1 g of dry extract for the fruits and 58.5 ± 0.01 mg of gallic acid per 1 g of dry extract for the leaves, as shown in Table 1. The tannin content in the investigated plant parts was shown to be at a very similar level for the stem and leaves (4.5 ± 0.0 mgPE/g of dry extract and 4.6 ± 0.0 mgPE/g of dry extract, respectively). The fruits appeared to possess a higher level of tannins—17.1 ± 0.04 mgPE/g of dry extract.

The TFC in ethanolic extracts determined by El-Zahar et al., 2022 was described as being at the level of 59.6 ± 1.3 mg QE/g, expressed per g of dry extract in leaves [18]. Khromykh et al., 2018 determined that the TFC was at the level of 1.4 ± 6.4 mg of rutin equivalents/g of wet weight in isopropanolic fruit extracts [30]. Okatan et al., 2018 described total flavonoid contents ranging from 2.6 to 965.9 mg CAT/g of fresh fruit weight, depending on the plant variety [31]. In our study, the level of TFC determined ranged between the levels given in the literature data with regard to the fruits. The highest TFC level we determined in leaves was 15.3 ± 0.1 mg of quercetin (Q) per 1 g of dry extract, while Dimitrijević et al., 2020 reported a TFC for leaves at the level of 1745 µg of rutin per mg of dry extract [29]. The values of the TFC in our study are shown in Table 1.

### 3.2. Quantitative and Qualitative Analysis of Phenolic Acids and Flavonoids in Individual Organs of Berberis vulgaris

**Figure 2:**
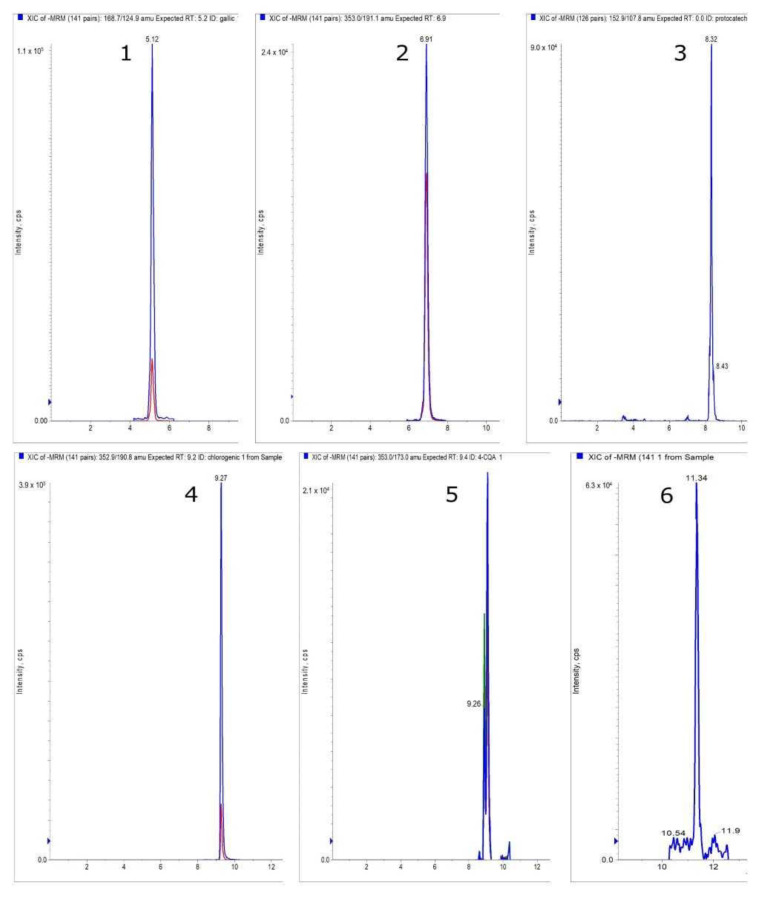
Extracted ion chromatograms of phenolic acids detected in the *Berberis* stem, obtained in the multiple reaction monitoring (MRM) mode: 1—gallic acid; 2—3-caffeoylquinic acid; 3—protocatechuic acid; 4—5-caffeoylquinic acid; 5—4-caffeoylquinic acid; 6—caffeic acid.

In the next stage of the study, we performed LC-MS/MS quantitative and qualitative analysis of phenolic acids and flavonoids in the barberry leaves, fruits, and stem (Figure 2).

Gallic acid was found only in the stem at the level of 10.5 ± 0.2 ng/mg (0.0 ± 0.0002 mg/g), while Eroğlu et al. described high concentrations of gallic acid in fruits from different harvesting locations; they ranged between 41.19 ng/mg in a water extract and 330.40 ng/mg in an ethanolic extract [3]. Gholizadeh-Moghadam et al. also reported that barberry fruits contained 334.8 mg/L of gallic acid, expressed per L of fresh juice [32]. El-Zahar et al., 2022 determined gallic acid in leaves at the level 10.2 ± 0.07 mg/ml in an ethanolic extract [18]. Our findings differ from these and confirm the influence of geographical conditions. 3-caffeoylquinic acid was also found only in the stem at the level of 2.4 ± 0.1 ng/mg (0.0 ± 0.0001 mg/g), and this secondary metabolite was not previously determined in *Berberis vulgaris.* In this species, we determined protocatechuic acid in the barberry stem and fruits, with the level of 120.5 ± 6.4 ng/mg (0.12 ± 0.006 mg/g) in the fruits and the level of 54.9 ± 1.1 ng/mg (0.05 ± 0.001 mg/g) in the stem. We did not determine protocatechuic acid in the leaves, while El-Zahar et al., 2022 determined this acid in leaves at the level 2.3 ± 0.01 mg/mL in a liquid extract [18].

5-caffeoylquinic acid was determined in each part of the plant, with an exceptionally high level in the leaves: 2556.4 ± 47.6 ng/mg (2.6 ± 0.05 mg/g) and fruits 3049.2 ± 17.9 ng/mg (3.0 ± 0.02 mg/g). 4-caffeoylquinic acid was present in the fruits (25.7 ± 0.5 ng/mg (0.0 ± 0.0005 mg/g)) and leaves (25.1 ± 0.4 ng/mg (0.0 ± 0.0004 mg/g)). Caffeic acid was determined by Eroğlu et al. in fruits, with the highest level of 152.22 ng/mg in a water extract [3]. In turn, Gholizadeh-Moghadam et al. [32] reported a caffeic acid level of 51.8 mg/L in fresh fruit juice. El-Zahar et al., 2022 determined caffeic acid in leaves at the level of 9.5 ± 0.06 mg/mL in a liquid extract [18]. In our study, caffeic acid was present in each part of the plant. The determined values ranged between 105.3 ± 3.9 ng/mg (0.1 ± 0.004 mg/g) in the leaves and 252.7 ± 6.4 ng/mg (0.2 ± 0.006 mg/g) in the stem.

In the study conducted by Eroğlu et al., high concentrations of syringic acid were observed; they found 867.85 ng/mg of syringic acid in a fruit extract [3]. El-Zahar et al., 2022 also determined syringic acid in leaves at the level of 3.2 ± 0.02 mg/mL in a liquid extract [18]. In our study, fruits collected in Poland did not contain syringic acid, or it was found in a trace amount (concentration below LOQ—732 ng/mL), which indicates a relevant influence of climate conditions on plant metabolism and properties. The phenolic acid content in the individual organs is shown in Table 2.

The sums of the phenolic acids determined in this study were 0.5 mg/g for the stem, 3.4 mg/g for the fruits and 2.7 mg/g for the leaves. These values are lower than the determined tannin content and indicates that in these parts of *Berberis vulgaris* L. there are some additional phenolic acids, which we were not be able to determine using our standards. The tannin contents determined in this study were 4.5 ± 0.004 mgPE/g for the stem, 4.6 ± 0.004 mgPE/g for the leaves, and 17.1 ± 0.04 mgPE/g for the fruits (Table 1). The TPC values (57.7 ± 0.001 mgGAE/g for the stem, 58.5 ± 0.01 mgGAE/g for the leaves, and 52.8 ± 0.01 mgGAE/g for the fruits) also indicate that in addition to the phenolic acids there were some additional polyphenols not determined in this study, but the obtained data were consistent.

The Folin–Ciocalteu reagent should be viewed as a measure of total antioxidant capacity, not phenolic content, as it is significantly reactive with other compounds besides phenols, such as thiols, vitamins, amino acids, proteins, nucleotide bases, unsaturated fatty acids, carbohydrates, organic acids, inorganic ions, metal complexes, aldehydes, and ketones. Typically, the Folin–Ciocalteu test result gives an approximation of the total phenol content since phenols are the most abundant antioxidants in most plants [33]. However, in our studies, we showed discrepancies; therefore, this indicates the likely presence of compounds other than phenols, such as the mentioned thiols, vitamins, amino acids, proteins, nucleotide bases, unsaturated fatty acids, carbohydrates, organic acids, inorganic ions, metal complexes, aldehydes, and ketones.

When analysing flavonoid aglycones in *Berberis vulgaris* L., we identified catechin, luteolin, quercetin, and eriodictyol in the individual organs of *Berberis vulgaris* L. The fruits contained all four identified compounds and the highest levels of catechin (372.1 ± 0.7 ng/mg (0.4 ± 0.0007 mg/g)) and quercetin (19.4 ± 0.45 ng/mg (0.02 ± 0.0004 mg/g)). The leaves contained the greatest amount of luteolin (11.9 ± 0.3 ng/mg (0.0 ± 0.0003 mg/g)). The eriodictyol content ranged from 0.4 ± 0.01 ng/mg (0.00 ± 0.0001 mg/g) in the fruit to 3.9 ± 0.1 ng/mg (0.00 ± 0.0001 mg/g) in the stem. The flavonoid aglycone contents in the individual organs are shown in Table 2.

Among the flavonoid glycosides, we qualitatively and quantitatively determined eleutheroside E, eriodictyol-7-glucopyranoside, rutin, hyperoside, luteoloside, isoquercetin, narcissoside, isorhamnetin-3-glucoside, quercitrin, naringenin 7-glucoside, and afzelin. Eleutheroside E was present in the stem, with the content of 116.2 ± 1.9 ng/mg (0.1 ± 0.002 mg/g)). Rutin and isoquercitin were contained only in the leaves, but their contents were relatively high (263.4 ± 8.5 ng/mg (0.3 ± 0.008 mg/g) and 536.3 ± 10.9 ng/mg (0.5 ± 0.01 mg/g), respectively). Interestingly, rutin was determined in fruits by Nuralin and Gürü, 2022 at the level of 0.2 ± 0.01 mg/g [31].

Luteoloside was found only in the leaves and stem, with a higher level in the leaves (92.5 ± 0.7 ng/mg (0.1 ± 0.0007 mg/g)) and 11.4 ± 0.4 ng/mg (0.01 ± 0.0004 mg/g), respectively). Narcissoside and naringenin 7-glucoside were identified in the leaves and fruits in small amounts. Isorhamnetin-3-glucoside and afzelin were determined only in the fruits in amounts of 44.1 ± 0.3 ng/mg (0.04 ± 00.0003 mg/g) and 261.6 ± 0.4 ng/mg (0.3 ± 0.0004 mg/g), respectively. The fruits were also characterised by a very high content of quercitrin (1112.7 ± 21.7 ng/mg (1.1 ± 0.02 mg/g)), which was also present in the leaves and stem, but in much lower concentrations (9.5 ± 0.2 ng/mg (0.00 ± 0.0002 mg/g) and 8.9 ± 0.2 ng/mg (0.00 ± 0.0002 mg/g), respectively). We confirmed the presence of hyperoside in the leaves and fruits [32,33,34,35]. Additionally, this compound was detected in the stem. The fruits and leaves, however, contained relatively high amounts of hyperoside (282.9 ± 6.7 ng/mg (0.3 ± 0.007 mg/g)) and 1942.2 ± 47.4 ng/mg (1.9 ± 0.05 mg/g), respectively) compared to the stem, which contained 38.9 ± 1.1 ng/mg of the compound (0.04 ± 0.001 mg/g). The flavonoid glycoside contents in the individual organs are shown in Table 2.

### 3.3. Antiradical Activity of Individual Organs of Berberis vulgaris

This work evaluated the antioxidant activity of extracts obtained from the fruits, leaves, and stem. The DPPH analysis showed that the raw material extracts from the individual plant parts were characterised by very similar values ranging from 63.9 ± 0.001 mgTe/g for the stem to 65.2 ± 0.005 mgTe/g for the leaf extracts. The ORAC results showed that the leaves had the highest antioxidant activity, i.e., 267.8 ± 0.03 mgTE/g, followed by the fruits (215.2 ± 0.02 mgTE/g), and stem (167.7 ± 0.04 mgTE/g). The ABTS results also showed that the leaves had the highest antioxidant activity (140.5 ± 0.04 mgTE/g). The other values determined with the ABTS method were 122.9 ± 0.01 mgTE/g for the stem and 117.9 ± 0.01 mgTE/g for the fruits. The high antioxidant activity in the leaf and fruit extracts may be related to the highest content of polyphenols in these extracts compared to the other samples.

Aliakbarlu et al., 2018 described a significantly strong scavenging effect of a barberry fruit acetone extract, while an ethanol extract showed the lowest scavenging activity [2]. Eroğlu et al., 2020 reported the DPPH radical scavenging activity of barberry fruits in the range of 11.92–40.44% [3], while Motalleb et al. determined the DPPH scavenging activity of barberry fruits in the ranges of 82.52 ± 0.64% and 73.62 ± 1.87 for water and ethanol extracts %, respectively [28]. Gholizadeh-Moghadam et al. reported the antioxidant activity of *Berberis vulgaris* L. fruit extract at a level of 56.84% [33]. These authors generally highlight the higher antioxidant potential of water extracts [8]. Although fruits have attracted attention so far, Nova-Baza et al. (2022) showed that leaves are the most valuable part of *Berberis vulgaris* L. in terms of antioxidant and nutritional properties [36], as was also determined in our study. Other data from the literature indicate a weaker antioxidant potential of various extracts of the root or herb of *Berberis vulgaris*; Movahedi et al., 2018, e.g., determined the EC50 for *Berberis vulgaris* L. fruits at the level of 31.2 ± 0.5 μg/ml in a decoction of dried fruits [37]. The results obtained from the antiradical activity analysis are presented in Table 3.

### 3.4. Lipoxygenase Inhibitory Activity of Individual Organs of Berberis vulgaris

In traditional Iranian medicine, *Berberis vulgaris* L. is a known anti-inflammatory agent. In addition, traditional European medicine used *Berberis* bark to treat inflammation, especially in diseases of the digestive system. Recent studies have shown that the main anti-inflammatory mechanisms involve changes in the cell immune response to Th2, Treg induction, inhibition of inflammatory cytokines (IL-1, TNF, and IFN-γ), and stimulation of IL-4 and IL-10 [38]. However, the anti-inflammatory effect of individual parts of the plant has not been evaluated so far. In order to study the anti-lipoxygenase properties of *Berberis vulgaris*, an experiment with one of the enzymes involved in the development of inflammation was carried out. Therefore, the direct ability of the extracts to inhibit lipoxygenase (LOX) activity was investigated. Table 4 shows the percentage of LOX inhibition for the concentrations of 1 mg dry extract/mL reaction mixture. The stem and leaf extracts were shown to have higher LOX inhibitory activity (83.56% and 79.78% LOX inhibition, respectively). The weakest effect (45.24% of inhibition) was exerted by the fruit extract. These data are of great interest as the stems show the greatest inhibition of LOX against fruits and leaves, despite having low levels of both phenolic acids and flavonoids. It should be emphasized that, as in the case of the antioxidant capacity, there are probably compounds involved in the anti-inflammatory effect other than those tested here. However, the results of the chromatographic analysis showed the presence of eleutheroside E in the stem, which distinguishes this organ from the others. It is also known that the raw material contains berberine—an alkaloid with a proven anti-inflammatory effect, but its content has not been thoroughly investigated in relation to individual parts of the plant [39]. For example, El-Zahar et al., 2022 determined berberine in leaves at the level of 18.8 ± 0.2 mg/mL in a liquid extract [18]. This analysis again indicates that further research into the phytochemical composition of *Berberis Vulgaris* L. is very important.

As shown in Table 5, the total content of polyphenols positively correlates with the antioxidant activity of the tested extracts. However, negative correlations were found between the antioxidant activity and the content of phenolic acids. The result concerns both the sum of the determined phenolic acids and the individual compounds of this group. We also found a negative correlation between tannins and the antioxidant capacity of the tested extracts. This result suggests that groups of compounds other than polyphenols are responsible for the high antioxidant activity. This confirms our observations regarding the Folin–Ciocalteu reaction, which was discussed above, and further justifies further research on the presence of other antioxidants in *Berberis vulgaris* L. One of the important antioxidants determined in *Berberis vulgaris* L. is vitamin C, but the data relate only to fruit. For example, Akbulut et al. described the content of 25 mg/100 g of fresh fruit weight [40], while Ardestani et al., 2015 described 16.373 mg/100 g of fresh fruit extract [41].

On the other hand, the total content of flavonoids and the chromatographically determined sum of flavonoids correlated positively with the antioxidant activity of the tested extracts. In addition, most of the identified flavonoids also individually correlated positively with the antioxidant activity. Positive correlations were found with luteolin, eriodictiol, hyperoside, luteoloside, naringenin-7-glucoside, narcissoside, and quercitrin. This result suggests that flavonoids are one of the groups of secondary metabolites of *Berberis Vulgaris* L. responsible for the high antioxidant potential of the tested parts of the plant. On the other hand, a similar profile of antioxidant activity with simultaneous differences in the chemical composition of the flavonoid content, despite positive correlations, again suggests that there are other compounds responsible for the antioxidant activity than those examined here.

Our research confirms that *Berberis vulgaris* L. is a highly chemically rich raw material. Its anti-inflammatory effect has not yet been thoroughly studied. In our study, we only assessed inhibitory activity against an inflammation-related enzyme, and this should be considered a preliminary study to facilitate further research. Correlation analysis shows that LOX inhibitory activity is positively correlated with TPC. However, here, as in the case of antioxidant activity, the correlation with phenolic acids was negative. So, the presence of other responsible compounds with LOX inhibitory activity can be assumed. However, in the light of our research, the issue of flavonoids seems to be equally important. On the other hand, the correlation analysis of TFC, the sum of the flavonoids, and the individual flavonoids showed that they correlate positively with LOX inhibitory activity. This is important because the content profile of the tested flavonoids varied significantly between the organs. And although the total flavonoids in the stem were the lowest, this organ showed the highest percentage of LOX inhibition. The flavonoids that positively correlated with the percentage of LOX inhibition were luteolin, eriodictyol, hyperoside, luteoloside, naringenin-7-glucoside, narcissoside, and quercitrin. However, when comparing the ability to inhibit LOX, it can be seen that the higher content of these compounds in leaves and fruits compared to those of the stem does not give these organs an advantage over the stem in terms of ability to inhibit LOX. When analysing the chemical composition, we noticed that the stem differed primarily in the presence of eleutheroside E (116.2 ± 1.9 ng/mg). The literature data indicate the anti-inflammatory activity of eleutheroside E, which puts the *Berberis* stem in a new light and makes it a valuable plant material [42].

**Figure 3:**
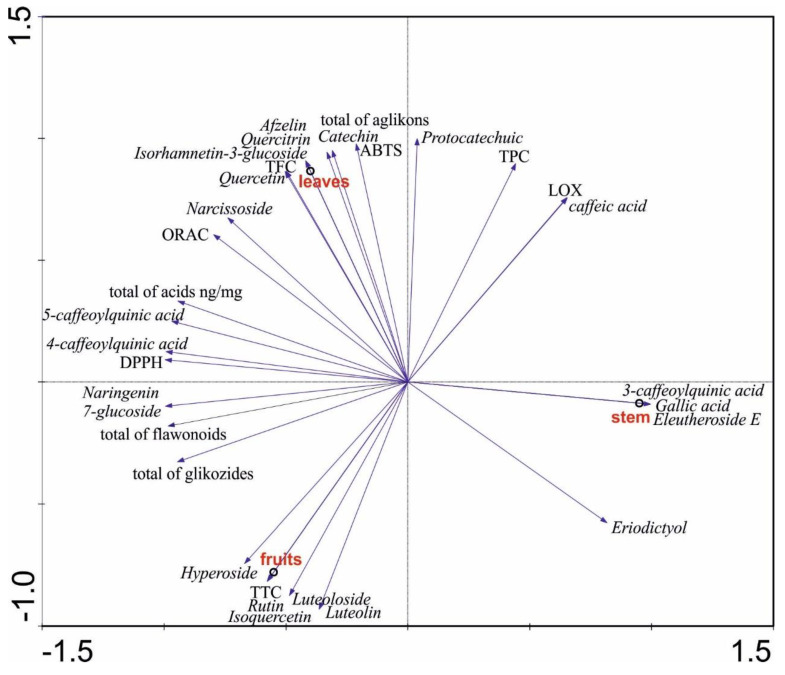
Principal component analysis of chemical components of investigated phenolic acids, flavonoid aglycones, and flavonoid glycosides in the barberry extracts. Analysis was performed using Statistica 6.0.; length of gradient of 1st axis, 1.294; cumulative percentage variance of data of 1st axis, 82.5; cumulative percentage variance of data of 2nd axis, 17.5.

Figure 3 shows the PCA analysis of the chemical composition of the leaves, fruits, and stem of *Berberis vulgaris* L. as well as the analysis of the TPC, TFC, and TTC and the analysis of the obtained results from the analysis of the antioxidant activity—ABTS, ORAC, and DPPH. The analysis illustrates what has been described about the correlation. Once again, it is seen that the stem differs from the other organs in terms of secondary metabolite content and LOX inhibitory activity, which again suggests the presence of chemical compounds responsible for this activity other than those investigated. The analysis shows that the leaves are the richest in terms of the content of compounds determined in this study and that the leaves are characterized by the highest antioxidant activity. This indicates that the study of leaves for the content of phenolic acids and flavonoids is a direction worth continuing, while other chemical groups should be searched for in the stem.

## 4. Conclusions

*Berberis vulgaris* L. is a species with high antioxidant potential. All three methods for the determination of the antioxidants indicate that the genus has high and similar antioxidant activity. This study confirms the high antioxidant potential of barberry fruit, but the tests used have shown that the leaves are a more valuable part than the fruits or the stem in this respect. The content of phenolic acids, flavonoid glycosides, and flavonoid aglycones in *Berberis vulgaris* L. varies depending on the organ, with the highest amount of polyphenols present in the leaf and fruit extracts. The leaves also happened to be the richest source of flavonoids. The chemically rich, and so far underestimated, leaves turned out to be, along with the popular fruits, a valuable food and chemopreventive raw material. The present results also indicate that some other chemicals may be responsible for the antioxidant activity of *Berberis vulgaris* L. and that there is a great need to continue research on the content and influence of other bioactive compounds, including alkaloids, tannins and terpene derivatives, on the antioxidant activity of *Berberis*. The percentage of LOX inhibition indicates that *Berberis vulgaris* L. is the valuable plant material in this respect. It is dependent on the chemical composition of the part of plant and needs further research. The present results provide new and important knowledge on the chemical composition of *Berberis vulgaris* L., which is undoubtedly a valuable and noteworthy raw material with high antioxidant and chemopreventive potential.

## Figures and Tables

**Table 1:** Total content of polyphenols (TPC), total tannin content (TTC), and total flavonoid content (TFC) in the barberry extracts. TPC was calculated on the basis of the equation of the gallic acid standard curve and expressed in mg of gallic acid per 1 g of dry extract (GAE—equivalent gallic acid). TTC was calculated on the basis of the equation of the pyrogallol standard curve and expressed in mg of pyrogallol (PE) per 1 g of dry extract. TFC was expressed in mg of quercetin (Q) per 1 g of dry extract. All results were expressed as mean ± standard deviation (SD) of measurements in triplicate. Extraction efficiencies [EE] were expressed in % of raw plant material.

	EE	TPC	TTC	TFC
Stem	9.1	57.7 ± 0.001	4.5 ± 0.003	4.6 ± 0.01
Leaves	20.5	58.5 ± 0.01	4.6 ± 0.004	15.3 ± 0.1
Fruits	35.7	52.8 ± 0.01	17.1 ± 0.04	6.1 ± 0.02

**Table 2:** Phenolic acids, flavonoid aglycones, and flavonoid glycosides in the barberry extracts. Metabolite content expressed in ng/mg of dry extract. Mean values of three replicate assays with standard deviation. Abbreviations: <LOQ—below the quantification level—the metabolite was detected, but its concentration could not be determined; nd—not detected.

Sample	*Berberis* Stem	*Berberis* Fruits	*Berberis* Leaves
Phenolic acids			
Gallic acid	10.5 ± 0.2	<LOQ	nd
3-caffeoylquinic acid	2.4 ± 0.1	nd	<LOQ
Protocatechuic acid	54.9 ± 1.1	120.5 ± 6.4	nd
5-caffeoylquinic acid	175.6 ± 5.6	3049.2 ± 17.9	2556.4 ± 47.6
4-caffeoylquinic acid	0.4 ± 0.01	25.7 ± 0.5	25.1 ± 0.4
Caffeic acid	252.7 ± 6.4	237.4 ± 8.9	105.3 ± 3.9
Sum of phenolic acids	496.6	3432.9	2686.9
Flavonoid aglycones			
Catechin	46.7 ± 1.4	372.2 ± 0.7	nd
Luteolin	2.9 ± 0.2	0.3 ± 0.01	11.9 ± 0.3
Quercetin	<LOQ	19.4 ± 0.5	1.6 ± 0.1
Eriodictyol	3.9 ± 0.1	0.4 ± 0.01	1.6 ± 0.04
Flavonoid glycosides			
Eleutheroside E	116.2 ± 1.9	nd	nd
Eriodictyol-7-glucopyranoside	3.9 ± 0.1	0.4 ± 0.01	1.6 ± 0.04
Rutin	<LOQ	<LOQ	263.4 ± 8.5
Hyperoside	38.9 ± 1.1	282.9 ± 6.7	1942.2 ± 47.4
Luteoloside	11.4 ± 0.4	nd	92.5 ± 0.7
Isoquercetin	<LOQ	<LOQ	536.3 ± 10.9
Narcissoside	nd	46.3 ± 0.3	17.8 ± 0.4
Isorhamnetin-3-glucoside	nd	44.1 ± 0.2	<LOQ
Quercitrin	8.9 ± 0.2	1112.7 ± 21.7	9.5 ± 0.2
Naringenin 7-glucoside	nd	3.8 ± 0.1	4.5 ± 0.1
Afzelin	<LOQ	261.6 ± 0.4	<LOQ
Sum of flavonoids	229.0	2143.7	2881.4

**Table 3:** Antioxidant activity of *Berberis vulgaris* L. extracts. Antioxidant activity expressed as the DPPH• scavenging assay [expressed as mg Trolox (Trolox equivalents)/g of dry extract], antiradical capacity (ABTS+•) [expressed as mg Trolox (Trolox equivalents)/g of dry extract], and oxygen radical absorbance capacity (ORAC) [expressed as mg Trolox (Trolox equivalents)/g of dry extract]. Values are presented with the mean ± standard deviation of triplicate measurements.

Sample	ABTS [mgTE/g]	ORAC [mgTE/g]	DPPH [mgTE/g]
Stem	122.96 ± 0.01	167.70 ± 0.04	63.88 ± 0.001
Leaves	140.49 ± 0.04	267.81 ± 0.03	65.25 ± 0.005
Fruits	117.93 ± 0.01	215.25 ± 0.02	64.73 ± 0.008

**Table 4:** Inhibition of lipoxygenase (LOX) by *Berberis vulgaris* L. samples tested at concentrations of 1 mg of dry extract/mL of the reaction mixture. Mean values of three replicate assays with standard deviation. The subscript letters (a,b) indicate significant differences at *p* < 0.05.

Sample	% LOX Inhibition
Stem	83.6 ± 0.1 ^a^
Leaves	79.8 ± 2.2 ^a^
Fruits	45.2 ± 2.4 ^b^

**Table 5:** Pearson’s correlation coefficients between biological activities and concentrations in extracts of *Berberis vulgaris* L.

	ABTS	ORAC	DPPH	% LOX Inhibition
TPC	1	1	1	1
TFC	1	1	1	1
TTC	−1	−1	−1	−1
Sum of phenolic acids	−1	−1	−1	−1
Sum of Flavonoids	1	1	1	1
Protocatechuic acid	−1	−1	−1	−1
5-caffeoylquinic acid	−1	−1	−1	−1
4-caffeoylquinic acid	−1	−1	−1	−1
Caffeic acid	−1	−1	−1	−1
Catechin	−1	−1	−1	−1
Luteolin	1	1	1	1
Eriodictyol	1	1	1	1
Quercetin	−1	−1	−1	−1
Hyperoside	1	1	1	1
Luteoloside	1	1	1	1
Naringenin 7-glucoside	1	1	1	1
Narcissoside	1	1	1	1
Quercitrin	1	1	1	1

## Data Availability

Not applicable.

## Supplementary Materials

The following supporting information can be downloaded at: https://www.mdpi.com/article/10.3390/antiox12071467/s1, Table S1: Summary of optimized QTRAP parameters for the LC-MS analysis of phenolic acids and flavonoid compounds, Abbreviations: Q1/Q3—m/z values for precursor and fragment ion detected in Q1 and Q3 quadrupole, respectively (tracked MRM transitions); declustering potential (DP); entrance potential (EP); collision cell exit potential (CXP); collision energy (CE), Table S2: Analytical parameters used for quantitative determination of phenolic acids and flavonoids detected in samples.
