# Supplementary material for: Evaluation of the Antioxidant and Anti-Lipoxygenase Activity of Berberis vulgaris L. Leaves, Fruits, and Stem and Their LC MS/MS Polyphenolic Profile
Source: Antioxidants (Basel). 2023 Jul 21;12(7):1467. doi: 10.3390/antiox12071467 (PMC10376855; doi:10.3390/antiox12071467)
Supplement: Supplementary file 1 [file antioxidants-12-01467-s001.zip › antioxidants-2464651-supplementary.pdf]

Table S1. Summary of optimized QTRAP parameters for the LC-MS analysis of phenolic acids and flavonoid compounds. Abbreviations: Q1/Q3 – m/z values for precursor and fragment ion detected in Q1 and Q3 quadrupole, respectively (tracked MRM transitions); declustering potential (DP); entrance potential (EP); collision cell exit potential (CXP); collision energy (CE).

Compound	Retention time [min]	Q1/Q3 [m/z]	DP [V]	EP [V]	CEP [V]	CE [eV]
**Phenolic acids**						
Gallic acid	5.16	168.7/78.9<br>168.7/124.9	-35<br>-35	-3<br>-3	-12<br>-12	-36<br>-14
3-Caffeoylquinic acid	6.93	352.9/191.1<br>352.9/178.9	-25<br>-25	-10<br>-10	-24.7<br>-24.7	-28<br>-22
Protocatechuic acid	8.42	152.9/80.9<br>152.9/107.8	-55<br>-55	-1<br>-1	-10<br>-10	-26<br>-38
5-Caffeoylquinic acid	9.24	353.0/190.9<br>353.0/85	-35<br>-35	-4.5<br>-4.5	-16<br>-16	-20<br>-60
4-Caffeoylquinic acid	9.38	352.9/173<br>352.9/135<br>352.9/179	-25<br>-25	-10<br>-10	-24.7<br>-24.7<br>-24.7	-21<br>-36<br>-22
4-Hydroxybenzoic acid	10.84	136.8/92.9	-30	-7	-10	-18
Gentisic acid	11.37	152.8/80<br>152.8/96.9	-70<br>-70	-4<br>-4	-16<br>-16	-110<br>-52
Caffeic acid	11.38	178.7/88.9<br>178.7/134.9	-30<br>-30	-6.5<br>-6.5	-12<br>-12	-46<br>-16
Vanillic acid	11.41	166.8/107.9<br>166.8/123	-35<br>-35	-4<br>-4	-12<br>-12	-18<br>-12
Syringic acid	11.42	196.9/122.8<br>196.9/181.9	-30<br>-30	-9<br>-9	-12<br>-12	-24<br>-12
3-Hydroxybenzoic acid	12.12	136.8/93<br>136.8/75	-35<br>-35	-4<br>-4	-16.7<br>-16.7	-16<br>-48
4-Hydroxycinnamic acid (*p*-coumaric acid)	14.10	162.7/119<br>162.7/93	-30<br>-30	-8<br>-8	-12<br>-12	-14<br>-44
Sinapic acid	14.47<br>14.94	222.8/121<br>222.8/148.9	-35<br>-35	-8.5<br>-8.5	-10<br>-10	-36<br>-20
Ferulic acid	14.80<br>15.22	192.8/133.9<br>192.8/177.9	-25<br>-25	-11.5<br>-11.5	-14<br>-14	-16<br>-12
3-Hydroxycinnamic acid (*m*-coumaric acid)	15.50	162.7/119<br>162.7/91	-35<br>-35	-4.5<br>-4.5	-12<br>-12	-14<br>-36
Rosmarinic acid	15.91	358.7/160.8<br>358.7/196.8	-50<br>-50	-5<br>-5	-26<br>-26	-20<br>-22
2-Hydroxycinnamic acid (*o*-coumaric acid)	16.80	162.7/119<br>162.7/93	-25<br>-25	-5<br>-5	-10<br>-10	-14<br>-46
Salicylic acid	17.91	136.8/93<br>136.8/75	-35<br>-35	-4<br>-4	-10<br>-10	-16<br>-48
**Flavonoid aglycones**						
Catechin	9.64	288.8/244.9<br>288.8/109	-45<br>-45	-4.5<br>-4.5	-16<br>-16	-16<br>-32

Epigallocatechin gallate	11.20	457/169.1	-25	-10	-28.6	-30
		457/125	-25	-10	-28.6	-30
Dihydromyricetin	12.10	319/193	-25	-10	-23.5	-30
		319/125	-25	10	-23.5	-30
Naringenin	14.52	270.8/119	-50	-11.5	-12	-34
		270.8/150.9	-50	-11.5	-12	-22
Taxifolin	15.15	302.7/124.9	-45	-3.5	-18	-26
		302.7/284.8	-45	-3.5	-18	-14
Myricetin	16.57	316.7/136.9	-55	-9	-14	-32
		316.7/150.9	-55	-9	-14	-26
Luteolin	17.82	284.7/132.9	-75	-9	-18	-38
		284.7/150.9	-75	-9	-18	-26
Eriodictiol	17.89	286.7/134.9	-45	-6	-12	-32
		286.7/150.9	-45	-6	-12	-18
Laricitrin (3'-O-Methylmyricetin)	17.9	330.97/151	-25	-10	-23.9	-30
		330.97/315.9	-25	10	-23.9	-30
Quercetin	17.94	300.7/150.9	-60	-2.5	-12	-26
		300.7/178.8	-60	-2.5	-12	-20
3-*O*-Methylquercetin	18.11	314.7/299.8	-55	-9.5	-22	-18
		314.7/270.8	-55	-9.5	-22	-26
Apigenin	18.64	268.8/117	-70	-9.5	-12	-44
		268.8/106.8	-70	-9.5	-12	-34
Kaempferol	18.85	284.7/116.8	-70	-5	-12	-46
		284.7/93	-70	-5	-12	-52
Isorhamnetin	18.99	314.7/299.7	-65	-2.5	-26	-20
		314.7/150.9	-65	-2.5	-26	-30
Isokaempferide	19.16	298.8/283.9	-50	-4.5	-12	-18
		298.8/226.9	-50	-4.5	-12	-28
Rhamnetin	20.10	314.7/165	-60	-5.5	-18	-24
		314.7/120.9	-60	-5.5	-18	-36
Sakuranetin	21.67	284.7/118.9	-60	-5.5	-12	-34
		284.7/164.8	-60	-5.5	-12	-20
Chrysin	21.82	252.8/208.9	-80	-10	-14	-22
		252.8/142.9	-80	-10	-14	-26
Prunetin	21.98	282.8/267.7	-55	-12	-18	-20
		282.8/238.7	-55	-12	-18	-26
Rhamnazin	22.37	328.7/270.8	-70	-3	-28	-26
		328.7/313.8	-70	-3	-28	-14
**Flavonoid glycosides**						
Eleutheroside E	10.38	740.8/416.9	-60	-3.5	-54	-36
		740.8/578.9	-60	-3.5	-54	-14
Luteolin 3′,7′-diglucoside	11.28	609.1/285	-70	-7.5	-28	-50
		609.1/447	-70	-7.5	-28	-32
Quercetin 3,7-dirhamnoside	11.35	592.8/445.7	-90	-4	-26	-48
		592.8/298.9	-90	-4	-26	-34
Eriodictyol-7-*O*-rutinoside (Eriocitrin)	11.93	594.8/286.9	-75	-4.5	-28	-34
		594.8/150.9	-75	-4.5	-28	-46
Quercetin-3-*O*-rutinoside (Rutin)	11.99	608.7/299.6	-90	-8	-30	-46
		608.7/270.9	-90	-8	-30	-60

Kempferol 3,7-dirhamnoside (Kaempferitrin)	12.16	576.8/284.8<br>576.8/430.9	-80<br>-80	-4.5<br>-4.5	-28<br>-28	-42<br>-30
Apigenin – 6-*C*-glucoside (Isovitexin)	12.38	430.8/310.9<br>430.8/340.9	-65<br>-65	-4.5<br>-4.5	-18<br>-18	-28<br>-26
Apigenin – 8-*C*-glucoside (Vitexin)	12.40	430.8/310.9<br>430.8/340.9	-75<br>-75	-4.5<br>-4.5	-20<br>-20	-26<br>-34
Quercetin-3-*O*-galactoside (Hyperoside)	12.80	462.7/299.7<br>462.7/254.7	-70<br>-70	-4<br>-4	-18<br>-18	-28<br>-42
Luteolin-7-*O*-glucoside (Luteoloside)	12.87	446.8/284.8<br>446.8/132.9	-70<br>-70	-10.5<br>-10.5	-20<br>-20	-30<br>-78
Quercetin-3-*O*-glucoside (Isoquercetin)	13.00	462.7/299.7<br>462.7/270.7	-85<br>-85	-1.5<br>-1.5	-20<br>-20	-30<br>-44
Eriodictyol-7-*O*-glucopyranoside	13.06	448.8/286.9<br>448.8/134.9	-75<br>-75	-4.5<br>-4.5	-20<br>-20	-24<br>-48
Kaempferol – 3-*O*-rutinoside (Nicotiflorin)	13.31	592.7/284.8<br>592.7/226.7	-65<br>-65	-12<br>-12	-30<br>-30	-38<br>-68
Isorhamnetin-3-*O*-rutinoside (Narcissoside)	13.52	622.8/314.9<br>622.8/298.8	-90<br>-90	-4.5<br>-4.5	-30<br>-30	-40<br>-52
Naringenin-7-*O*-rutinoside (Narirutin)	13.80	578.9/270.8<br>578.9/118.9	-90<br>-90	-4.5<br>-4.5	-24<br>-24	-34<br>-76
Naringenin-7-*O*-rhamnosidoglucoside (Naringin)	14.50	579.1/151<br>579.1/271	-80<br>-80	-4<br>-4	-26<br>-26	-54<br>-42
Kaempferol – 3-*O*-glucoside (Astragalin)	14.66	446.7/226.8<br>446.7/254.8	-75<br>-75	-9<br>-9	-20<br>-20	-54<br>-40
Isorhamnetin-3-glucoside	14.76	476.8/313.9<br>476.8/270.9	-95<br>-95	-10<br>-10	-22<br>-22	-30<br>-44
Quercetin 3-*O*-rhamnoside (Quercitrin)	14.83	446.7/299.7<br>446.7/270.7	-65<br>-65	-9<br>-9	-18<br>-18	-30<br>-40
Apigenin 7-*O*-glucoside	14.91	430.7/267.7<br>430.7/116.9	-70<br>-70	-9<br>-9	-20<br>-20	-38<br>-84
Naringenin 7-*O*-glucoside	15.12	432.7/270.8<br>432.7/118.9	-40<br>-40	-8.5<br>-8.5	-20<br>-20	-22<br>-64
Afzelin (Kaempferol 3-rhamnoside)	15.9	431.1/284.9<br>431.1/254.9	-25<br>-25	-10<br>-10	-27.6<br>-27.6	-30<br>-30
Tiliroside	17.39	592.8/284.8<br>592.8/254.7	-70<br>-70	-7.5<br>-7.5	-24<br>-24	-38<br>-56

Table S2. Analytical parameters used for quantitative determination of phenolic acids and flavonoids detected in samples.

Compound	LOD [ng/mL]	LOQ [ng/mL]	R^2	Linearity range [ng/mL]
**Phenolic acids**				
Gallic acid	900	1600	0.9988	1600-16000
3-Caffeoylquinic acid	50	150	0.9992	150-15000
Protocatechuic acid	200	400	0.9988	1890-18900
5-Caffeoylquinic acid	75	180	0.9991	180-18000
4-Caffeoylquinic acid	60	120	0.9990	150-15000
Caffeic acid	195	389	0.9991	389-19500
Syringic acid	500	732	0.9993	732-18300
**Flavonoid aglycones**				
Catechin	250	330	0.9982	330-6600
Luteolin	6	16	0.9974	33-1650
Eriodictyol	33	66	0.9984	66-6600
Quercetin	66	132	0.9977	132-6600
Apigenin	15	22	0.9979	89-4470
Isorhamnetin	300	480	0.9993	850-5100
Sakuranetin	70	140	0.9991	140-6200
**Flavonoid glycosides**				
Eleutheroside E	275	550	0.9989	1000-20000
Luteolin 3′,7′-diglucoside	250	500	0.9990	1250-25000
Rutin	150	300	0.9983	750-15000
Hyperoside	160	250	0.9983	500-25000
Luteoloside	50	100	0.9980	250-25000
Isoquercetin	160	250	0.9988	500-50000
Eriodictyol-7-glucopyranoside	150	300	0.9991	600-24000
Nicotiflorin	70	140	0.9993	140-60000

Narcissoside	75	150	0.9989	200-25000
Astragalin	100	250	0.9990	750-25000
Isorhamnetin-3-glucoside	100	225	0.9986	750-35000
Quercitrin	50	100	0.9986	1000-25000
Apigenin 7-glucoside	100	250	0.9989	750-25000
Naringenin 7-*O*-glucoside	100	167	0.9987	250-25000
Afzelin	10	20	0.9973	30-6000
